# Systematic Localisation Based on History and Clinical Examination

**DOI:** 10.21315/mjms2024.31.6.17

**Published:** 2024-12-31

**Authors:** Muhamad Azuan Samsudin, Thaanesh Manokaran, Muhammad Syamim Ali, Atiqah Al Aqilah Jamaluddin, Darvena Pillay Sashideran, Heng Pei Ting, Heng Yu Wei, Chin Wen Xin, Muhammad Safuan Sabri, Chiun Pei Zhi, Muhammad Zikri Yussof, Prehmanraj Mariyapan, Jafri Malin Abdullah, Zamzuri Idris, Abdul Rahman Izaini Ghani, Ihfaz Ismail, Ang Soo Yee, Diana Noma Fitzrol

**Affiliations:** 1 Department of Neurosciences, School of Medical Sciences, Universiti Sains Malaysia, Kelantan, Malaysia; 2 Brain and Behaviour Cluster, School of Medical Sciences, Universiti Sains Malaysia, Kelantan, Malaysia; 3 Department of Neurosciences and Brain Behaviour Cluster, Hospital Universiti Sains Malaysia, Kelantan, Malaysia

**Keywords:** localisation, history taking, clinical examination, neurology

## Abstract

Localisation in neurology is an important step in determining the location of a neurological lesion based on history taking and clinical examination before confirmation with imaging. Lesions can vary from the cortical to the subcortical, brainstem, and spinal cord levels, in which every presentation and finding from the patient can provide a rough idea of where the pathology is located. A differential diagnosis can later be made according to the duration of the symptoms and the cumulative signs and symptoms presented. This is considered the most important step in managing neurological patients to ensure that no stone is left unturned in making an accurate diagnosis.

## Introduction

In neurology, key factors for provisional diagnoses are based not only on imaging tests but also on thorough history taking and full neurological examinations. In neurological cases, every patient presents with at least one major sign and symptom with the duration of the complaint. Next, a targeted physical examination follows for a full picture of the pathology. Combining both inputs will provide a few differential diagnoses that help the clinician order investigations to confirm the diagnosis. This article summarises and narrows down the complaints to specific localisations in neurology based on history taking and clinical examination. The summaries are presented as tables and flow charts with narratives to aid in understanding localisation. Each sign and symptom must attain a certain level of localisation to aid the clinician in managing patients and improving their quality of life.

## Higher Mental Function Examination

**Figure 1:**
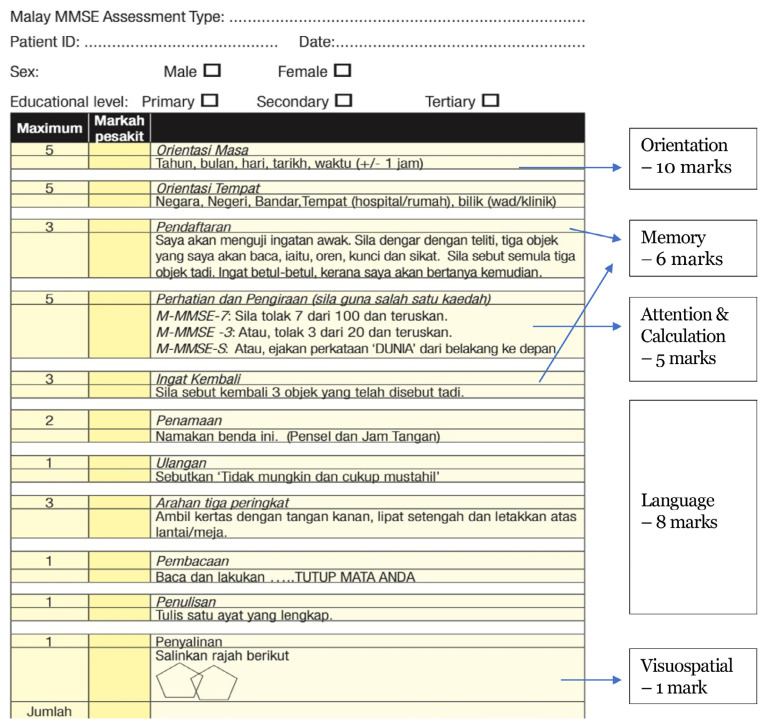
M-MMSE scores response

Three versions of the Malay-Mini Mental State Examination (M-MMSE) (1) have been validated in a group of Malay-speaking elderly population in Malaysia. Scores lower than the optimal cut-off scores indicate cognitive impairment, most commonly used to identify dementia as shown in Table 1 and description as Figure 1.

### Before MMSE

Level of consciousness: alertness and orientation.

Educational level: primary, secondary, diploma, and higher studies (degree and above) shown in Table 2.

### Handedness: Right or Left

Edinburgh Handedness Test (10 items):

- writing
- drawing
- throwing
- scissors
- toothbrush
- spoon
- knife (without fork)
- broom (upper hand)
- strike a match
- opening a box (lid)

Handedness determines the dominancy of cerebral hemispheres, although in 90%–95% of the population, the left cerebral hemisphere is dominant.

### Domain of Function with Localisation

#### Dominant Hemisphere (Left)

##### Orientation – time and place

E.g.: *Beritahu saya tarikh hari ini – tahun, bulan, tarikh, hari, masa.*

*Kamu berada di mana sekarang? – negara, negeri/mukim, bandar, bangunan, tingkat/bilik* Localisation

- Frontal (anterior cingulate gyrus), parietal (posterior cingulate gyrus), temporal (parahippocampal gyrus) – long term memory and memory retrieval.
- 1 + occipital (calcarine, cuneus, and lingual gyrus) with parietal (superior parietal lobule) – visual perception and integration of visuospatial info.

##### Registration – short term memory

E.g.: *Saya akan menguji ingatan awak. Tiga objek saya bacakan, oren, kunci, sikat.*

*Sila sebut semula tiga objek tadi. Saya akan bertanya kemudian.*

Localisation

- Temporal (Wernicke) – superior temporal sulcus and posterior portion of superior temporal gyrus.
- Frontal (Broca) – inferior frontal sulcus, triangular and opercular parts of inferior frontal gyrus.

##### Attention and calculation

E.g.: *Sila tolak 7 dari 100 dan teruskan. Sila tolak 3 dari 20 dan teruskan.*

*Sila eja kan “DUNIA” dari belakang ke depan.*

Localisation

- Frontal (inferior frontal sulcus, middle frontal gyrus, and anterior cingulate gyrus), parietal (posterior cingulate gyrus) – attention.
- Parietal (inferior parietal lobule – supramarginal and angular gyri) – calculation.

##### Delayed recall – long term memory

E.g.: *Sila sebut kembali 3 objek yang disebut tadi.*

Localisation

- Frontal (anterior cingulate gyrus), parietal (posterior cingulate gyrus), temporal (parahippocampal gyrus) – long term memory and memory retrieval.

##### Language: naming

E.g.: *Namakan benda ini: pensel, jam tangan.*

Localisation

- Occipital (visual perception).
- Temporal (superior and inferior temporal sulci, superior, middle and inferior temporal gyri) – language and auditory.
- Parietal (superior parietal lobule) – integration of visuospatial info.

##### Language: repetition

E.g.: *Sila sebut: “Tidak mungkin dan cukup mustahil”.*

Localisation

- Temporal (Wernicke) – superior temporal sulcus and posterior portion of superior temporal gyrus.
- Frontal (Broca) – inferior frontal sulcus, triangular and opercular part of inferior frontal gyrus.

##### Language: three-step command – apraxia

E.g.: *Ambil kertas dengan tangan kanan, lipat setengah dan letakkan atas meja.*

Localisation

- Occipital (visual perception).
- Temporal (superior and inferior temporal sulci and superior, middle and inferior temporal gyri) – language and auditory.
- Premotor cortex (anterior region of precentral gyrus) – programming activity.
- Frontal (superior and inferior frontal sulci, superior and middle frontal gyri) – motor execution.

##### Reading and comprehension

E.g.: *Baca dan lakukan arahan yang ditulis di ayat kertas ini.*

Localisation

- Occipital – visual perception.
- Temporal (superior temporal sulcus and gyrus) – comprehension.
- Parietal (superior parietal lobule) – integration of visuospatial info.

##### Writing

E.g.: *Tulis satu ayat yang lengkap.*

Localisation

- Occipital – visual perception.
- Temporal (superior temporal sulcus and posterior portion of superior temporal gyrus) – comprehension.
- Frontal (superior and inferior frontal sulci, superior and middle frontal gyri) – motor execution.

#### Non-dominant Hemisphere (Right)

##### Copying – apraxia

**Figure 2:**
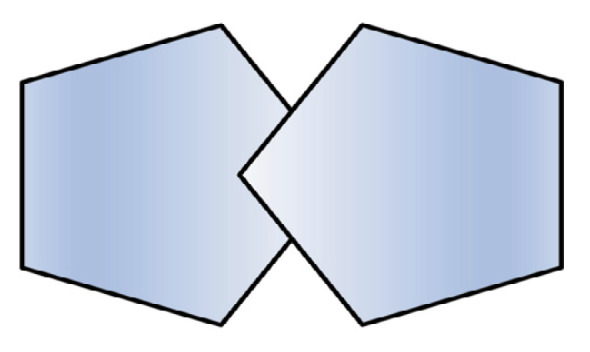
Image used in M-MMSE scoring activity

E.g.: *Salinkan rajah berikut.* (Figure 2)

Localisation

- Occipital – visual perception.
- Parietal (superior and inferior parietal lobules) – construct and gestalt.
- Frontal (superior and inferior frontal sulci and superior and middle frontal gyri) – motor execution.

## Approaches to Seizure Localisations Based on History Taking

In history taking, the demography of patients and epidemiology play a role, for example, age, sex, comorbidities, previous history of trauma, febrile illness and seizures, particularly in younger age groups (infant and paediatric).

**Figure 3:**
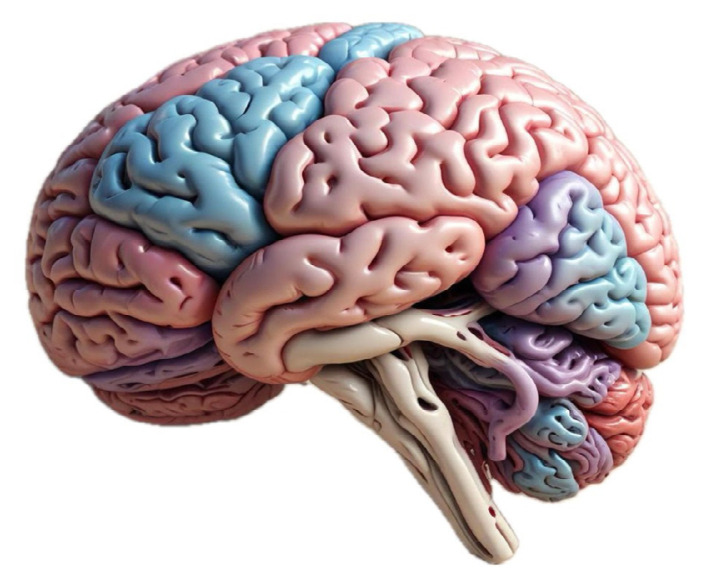
Human brain and divisions of the lobes

Seizure is defined as abnormal high firing synchronous neurons electrical activities with associations of different emotions, movement and behavioural changes (2). Figure 3 shows image of human brain and divisions of the lobes.[Fig f4a-17mjms3106_bc][Fig f4b-17mjms3106_bc]

## History Taking in Patients with Memory Impairment

Cognitive impairment is a transitional state between normal cognitive function and the presence of clinical symptoms of dementia. The decline in memory or other cognitive functions, including executive function, attention, language, and visuospatial skills are the most typical symptoms of cognitive impairment. Although patients with cognitive impairment may return to the normal state of cognitive function, most patients would have higher risk of dementia, disability and higher mortality rate. Despite the identification of cognitive impairment and dementia as public health problems incurring substantial economic burden on individuals, families, and countries, early diagnosis is often uncommon and public awareness is poor.

### Demography

The prevalence of cognitive impairment in Malaysia ranges from 11.0%–22.4%, which is comparable to other populations (6). The prevalence of cognitive impairment varies greatly across publications because of differences in definitions, diagnostic criteria, and sampling and assessment methods (6–8). It is however projected that the number would further increase considering the substantial increase in the proportion and number of the older population in Malaysia. In the Malaysian Clinical Practice Guidelines (CPG) dementia (3rd edition) (9), the assessment criteria for mild and major neurocognitive disorder are described subsequently.

### Diagnostic Criteria for Mild Neurocognitive Disorders (10)

Evidence of modest cognitive decline from a previous level of performance in one or more cognitive domains (complex attention, executive function, learning and memory, language and perceptual – motor or social cognition) based on the following:

- Concern of the individual, a knowledgeable informant or the clinician that there has been a mild decline in cognitive function.
- A modest impairment in cognitive performance, preferably documented by a standardised neuropsychological testing or in its absence, another quantified clinical assessment.
- The cognitive deficits do not interfere with capacity for independence in everyday activities (that is, complex instrumental activities of daily living, such as paying bills or managing medications are preserved, but greater effort, compensatory strategies or accommodation may be required).
- The cognitive deficits do not occur exclusively in the context of a delirium.
- The cognitive deficits are not better explained by another mental disorder (for example, major depressive disorder or schizophrenia).

**Figure 5:**
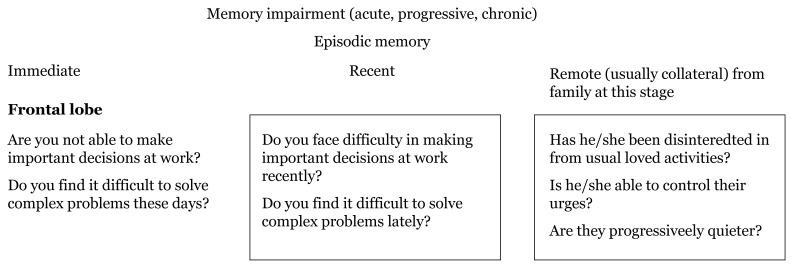
Questions for assessing types of memory impairment and cognitive symptoms related to specific location

**Figure 6:**
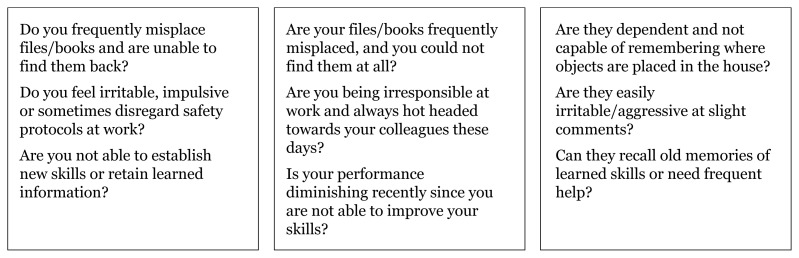
Questions for assessing types of memory impairment and cognitive symptoms related to specific location

**Figure 7:**
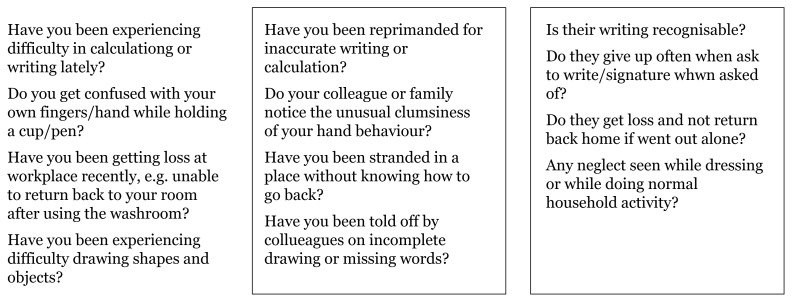
Questions for assessing types of memory impairment and cognitive symptoms related to specific location

**Figure 8:**
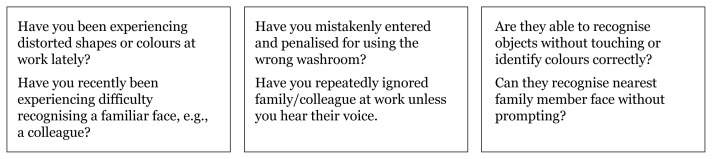
Questions for assessing types of memory impairment and cognitive symptoms related to specific location

Questions can be applied to our population to assess types of memory impairment based on the functions of different brain lobes and their representative localisation landmarks shown in Figures 5–8.

### Frontal Convexity (Dorsolateral) – Apathy, Indifference and Poor Abstract Thoughts

#### Superior Frontal Cortex Boundaries

Front marginal sulcus (anterior), precentral sulcus (posterior), superior frontal sulcus (lateral) and frontomarginal sulcus, orbitofrontal cortex and gyrus rectus (inferior).

#### Middle Frontal Cortex Boundaries

Frontomarginal sulcus and lateral orbital sulcus (anterior), precentral sulcus (posterior), inferior frontal sulcus (inferior and posterior), superior frontal sulcus (superior) and line connecting deepest point of inferior frontal sulcus and superior frontal sulcus.

#### Inferior Frontal Cortex Boundaries

Lateral orbital sulcus (anterior), precentral sulcus (posterior), and the lateral orbital sulcus and circular sulcus of insula (inferior) and line connecting the deepest points of inferior frontal sulcus with the deepest point of precentral sulcus (medial).

### Medial Frontal – Akinetic, Incontinent, Sparse Verbal Output

#### Anterior Cingulate Gyrus Boundaries

Cingulate sulcus (anterior superior and inferior boundaries), paracentral sulcus (posterior boundary), and pericallosal sulcus (inferior and posterior boundaries).

### Orbitofrontal – Disinhibition, Poor Judgement and Emotional Lability

#### Orbital Gyri Exclude Rectus Gyrus Boundaries

Frontomarginal sulcus (anterior and lateral boundaries), lateral orbital sulcus and circular sulcus of insula (lateral boundary), and olfactory sulcus (medial boundary).

### Superolateral Surface (Superior, Middle and Inferior Gyri)

- The stem and posterior ramus of lateral sulcus mark the separation of temporal, frontal, and parietal lobes.
- Lateral sulcus – superior border of temporal lobe,
- Indented by superior and inferior temporal sulci differentiating the gyri.

### Auditory Cortex

- Cortical deafness or surrounding association areas result in difficulty hearing spoken words (dominant) or difficulty appreciating music/rhythm (nondominant) boundaries.

### Superior Surface (Floor of Lateral Sulcus)

- Marked by transverse temporal gyrus/Heschl’s gyrus (primary auditory cortex) and planum temporale (larger on the left in men), medially bounded by circular sulcus of insula.
- Anterior end of insula (limen insulae) continuous with stem of lateral sulcus with cortex of parahippocampal gyrus (anteromedial part) and medial frontal cortex (paraterminal gyrus) below rostrum of corpus callosum.
- Posterior part blends into parietal and occipital lobes.
- Preoccipital notch: indentation in inferior temporal gyrus, 3 cm anterior to occipital pole. Line from parietooccipital sulcus to preoccipital notch defines anterior border of occipital lobe (lateral aspect). Midpoint of this line horizontal line passing forward to lateral sulcus separates temporal from parietal lobe.

#### Inferior Surface

- Line connecting preoccipital notch with cortex immediately behind splenium of corpus callosum separates temporal from occipital cortex.

### Middle and Inferior Temporal Cortex

- Disturbance of memory/learning, disordered memory (post ictal amnesia).
- Occipitotemporal sulcus separates medial border of inferior temporal gyrus from lateral border of fusiform (medial occipitotemporal) gyrus.
- Medial to fusiform gyrus is collateral sulcus.
- Anterior end of collateral sulcus curves below temporal pole as rhinal sulcus.

#### Inferior Surface

- Medial to collateral sulcus, parahippocampal gyrus forms medial border of inferior surface of temporal lobe.
- Uncus is small projection of medial surface of anterior end parahippocampal gyrus; limbic lobe.

### Lateral Surface

Boundaries: posterior to central sulcus (anterior), superior to lateral fissure (inferior). Posterior end: first line run from tip of parietooccipital sulcus (medial aspect) to preoccipital notch (separate parietal from occipital lobe) and second is imaginary extension of lateral fissure (separate parietal and temporal lobe).

Main region: post central gyrus (PcG), superior parietal lobule (SPL) and inferior parietal lobule (supramarginal and angular gyri).

Lesions in the PcG (dominant or nondominant) – contralateral disturbance of cortical sensation, e.g.:

- disturbed postural and passive movement sensations;
- disturbance in accurate localisation of light touch;
- loss of one/two-point discrimination;
- astereognosis; and
- sensory inattention.

### Lesions in Inferior Parietal Lobule

Dominant – Gerstmann’s syndrome (right-left confusion, finger agnosia, acalculia, agraphia). Non-dominant – anosognosia, dressing apraxia, geographical agnosia, constructional apraxia.

### Medial Surface

Boundaries: superior to cingulate sulcus (separate from limbic lobe) and anterior to parietooccipital sulcus. Main region: posterior paracentral lobule – contiguous with PcG, ends at marginal limb of cingulate sulcus and precuneus – posterior to cingulate sulcus, extension of SPL and ends at parietooccipital sulcus.

### Lateral Surface

This is located behind the parietotemporal line which connects the preoccipital notch with the parietooccipital sulcus. Within the occipital lobe, the lateral occipital sulcus, which is an extension of the superior temporal sulcus, is observed on the lateral surface.

### Medial Surface

The anterior border comprises the parietooccipital sulcus and extends inferiorly beyond the calcarine sulcus, anteriorly, where the parietooccipital and calcarine sulci join to form isthmus of gyrus cinguli. The mesial part of the hemisphere between the base and calcarine sulcus is formed by the lingula and between the calcarine and parietooccipital sulcus by the cuneus.

### Basal Surface

This is located posteriorly to the imaginary temporoparietal line connecting the preoccipital notch and inferior extension of the parietooccipital sulcus. The basal surface of the occipital lobe is formed by the occipitotemporal gyrus and basal surface of the lingula, separated by the collateral sulcus.

Lesions may cause the following:

- Visual field hemianopia with macular sparing.
- Visual illusion (micropsia, macropsia) and distortion of shape prosopagnosia (disturbance of interpretation and naming).

### Speech —History Taking

**Figure 9:**
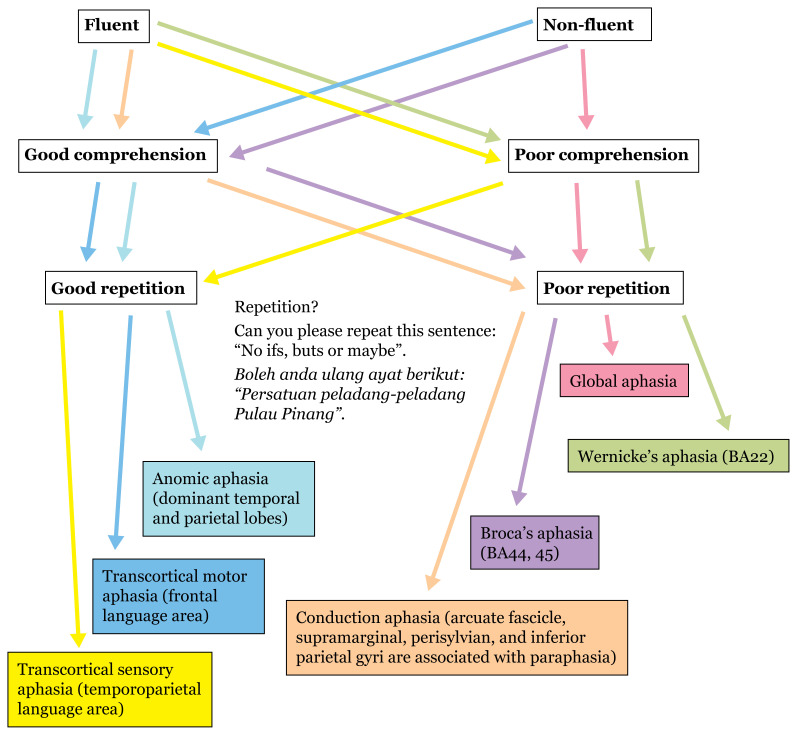
Types of aphasia

First, aphasia is categorised as fluent and nonfluent. Apart from fluency, comprehension and repetition further localise a lesion (see Figure 9).

## Types of Speech and Dysarthria

Dysarthria is mainly categorised according to symptom durations. Rule out whether this is associated with UMN/LMN or bulbar signs. Weakness of articulation muscles results not only from central nervous system lesions, such as stroke, but also from causes, such as demyelinating disease; therefore, this needs to be ruled out.[Fig f10-17mjms3106_bc]

### Spastic Dysarthria

- Characterised by increased muscle tone, stiffness and slow, effortful speech
- Speech may be strained or “strainedstrangled” in quality
- Lesion: UMN

### Flaccid Dysarthria

- Caused by weakness or paralysis of the muscles involved in speech production
- Speech may be breathy, weak, and imprecise
- Lesion: LMN

### Ataxic Dysarthria

- Characterised by incoordination and irregularity of speech movements
- Speech may be slurred, with irregular articulatory breakdowns
- Lesion: cerebellum

### Hypokinetic Dysarthria

- Associated with Parkinson’s disease
- Characterised by reduced range of movement, rapid rate, and reduced loudness
- Lesion: extrapyramidal

### Hyperkinetic Dysarthria

- Associated with conditions such as Huntington’s disease
- Characterised by involuntary movements, variable speech rate, and irregular articulation
- Lesion: extrapyramidal

## Approach to Dizziness

Dizziness is a term used to describe an array of subjective symptoms. It is necessary to further explore the experience felt by a patient to accurately narrow down the diagnosis. Dizziness can be categorised into four major groups; lightheadedness, pre-syncope, disequilibrium, and vertigo (11, 12).[Fig f11-17mjms3106_bc]

## Approaches to Facial Weakness and Pain

### Approach to Unilateral Facial Weakness (History Taking)

[Fig f12-17mjms3106_bc]

**Figure 12 f12-17mjms3106_bc:**
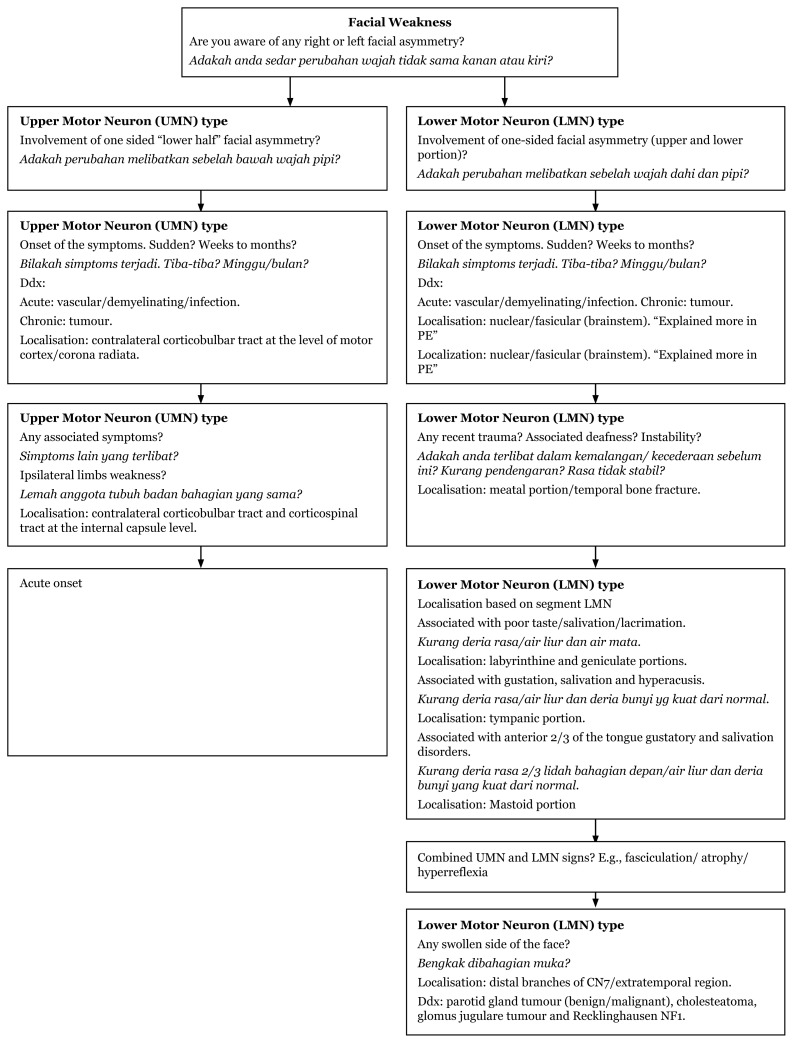
Unilateral facial weakness history taking and associated symptoms

### Approach to Unilateral Facial Weakness (Physical Examination)

[Fig f13-17mjms3106_bc] [Fig f14-17mjms3106_bc]

**Figure 13 f13-17mjms3106_bc:**
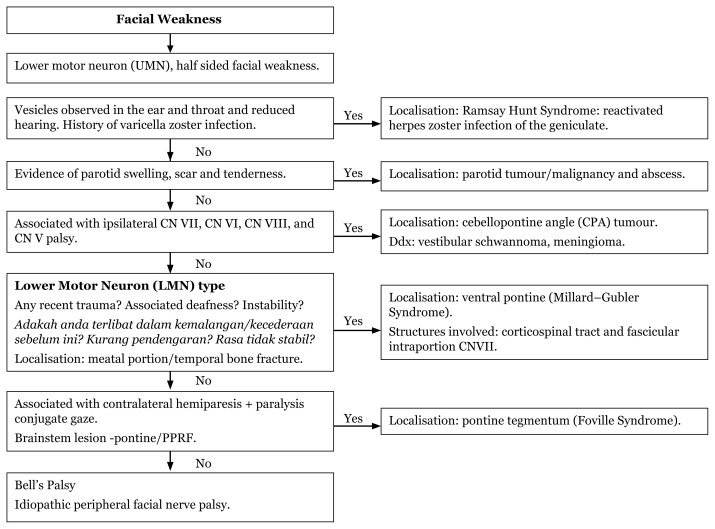
Unilateral facial weakness history taking and associated signs in LMN

**Figure 14 f14-17mjms3106_bc:**
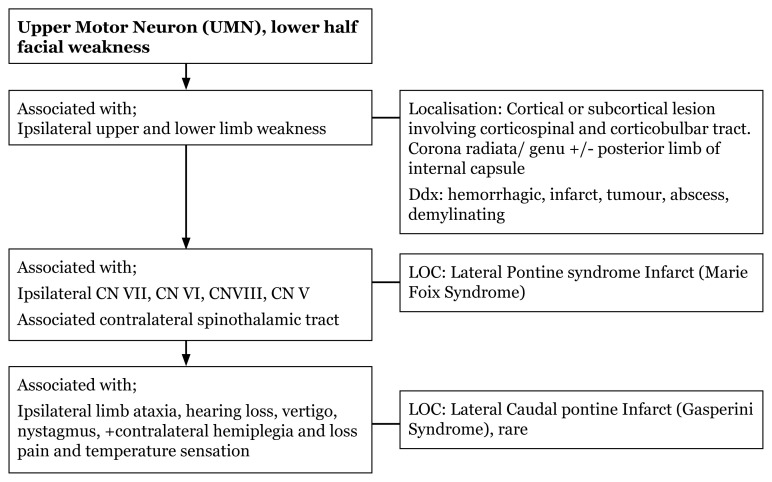
Upper Motor Neuron (UMN) localisation

### Approach to Unilateral Facial Pain

History taking:

- Onset acute/chronic
- Continuous or episodic
- Unilateral or bilateral
- Associated symptoms of headache, dental pain
- Any nasal obstruction/reduced sense of smell/taste
- Previous history of trauma, procedures or viral infection

[Fig f15-17mjms3106_bc]

In localising facial pain presenting complaints, we must stratify the onset of the pain – whether acute or chronic.

The characteristics of the facial pain – whether continuous or episodic, the nature – whether sharp shooting or burning, and the severity must be noted. The site where the facial pain started or involvement of the tongue/mouth must also be noted?

Then ask about recent nasal blockages. A history of foul smells may indicate sinusitis Dental pain that initially started as a toothache may indicate tooth infection. Pain aggravated by chewing movements or teeth clenching may indicate a temporomandibular disorder.

Is the facial pain precipitated by one-sided head pain with a prominent temporal artery – giant cell arthritis – or does the pain start only after the patient experiences a headache.

Recent dental procedures or trauma can also cause facial pain. Any association with the epiglottis, palatine tonsils, base of the tongue and/or posterior pharynx together with severe remitting type of pain must be investigated for trigeminal neuralgia.

Finally, if no cause of the facial pain is identified, it is most likely an idiopathic type which is characterised by continuous facial and/or teeth pain, sometimes varying in intensity throughout the day.

## Approach to Foot Drop

Corticospinal tract lesions usually present with weakness of unilateral hip flexion, knee flexion, and ankle dorsiflexion because the extensor muscles also receive innervation from the lateral vestibulospinal tract (14). While this weakness is not consistent with an isolated foot drop, ankle dorsiflexion weakness may be the primary symptom noticed by the patient (14). In addition to assessing subtle hip flexion and knee flexion weakness, a careful examination of other upper motor neuron signs, such as spasticity and hyperreflexia, should be performed.

In foot drop patients, the first movements to assess are ankle eversion and inversion. These movements need to be performed with the foot in a neutral or dorsiflexed position, which usually requires the examiner to dorsiflex the foot. If the foot remains plantarflexed, the gastrocnemius muscle can participate in eversion or inversion, potentially causing the examiner to mislocalise the lesion.

The common peroneal (fibular) nerve bifurcates into the deep peroneal and superficial peroneal nerves. The superficial peroneal nerve innervates the peroneus longus muscle that everts the foot and carries sensory information from the lower lateral leg and the dorsum of the foot (15). In this context, a patient with deep peroneal neuropathy would have a foot drop without ankle eversion weakness or obvious sensory loss. The only sensory fibres carried by the deep peroneal nerve convey sensation between the great and second toe, and thus this sensory distribution would need to be carefully assessed in a patient with foot drop but preserved eversion.

Common peroneal (fibular) neuropathy is a frequent cause of foot drop. The typical location of the lesion is where the common peroneal nerve crosses the head of the fibula. This frequently occurs in the context of excessive crossing of the legs, particularly in patients who have recently lost a significant amount of weight. A patient with common peroneal neuropathy will have numbness over the lower lateral leg and the dorsum of the foot and weakness of ankle dorsiflexion and eversion, but inversion will be normal because the tibialis posterior muscle that inverts the ankle is innervated by the tibial nerve.

L5 radiculopathy is another frequent cause of foot drop. The presence of radicular back pain suggests L5 radiculopathy. Upon examination, the presence of ankle inversion weakness distinguishes L5 radiculopathy from common peroneal neuropathy.

Sciatic neuropathy is a rare cause of foot drop (note that the term “sciatica” usually refers to L5 radiculopathy and not sciatic neuropathy). The sciatic nerve bifurcates into tibial and common peroneal nerves. A sciatic neuropathy can involve the fibres that travel in both of these nerves or preferentially involve the common peroneal nerve fibres. When both the tibial and common peroneal fibres in the sciatic nerve are affected, there is ankle plantarflexion (gastrocnemius muscle) weakness due to tibial nerve involvement, which would not be expected in common peroneal neuropathy. Involvement of the gastrocnemius muscle is also not seen with L5 radiculopathy, since the gastrocnemius muscle is innervated by the S1 nerve root. It is more difficult to distinguish sciatic neuropathy with preferential involvement of the common peroneal fibres from common peroneal neuropathy. In this case, the key factor is that the common peroneal nerve fibres contained within the sciatic nerve innervate the short head of the biceps femoris, part of the hamstring muscle. Involvement of this muscle, therefore, implicates the sciatic nerve and is inconsistent with common peroneal neuropathy. Because the hamstring is a very strong muscle, the examiner may not be able to appreciate the weakness of this muscle on examination. The presence of denervation of the short head of the biceps femoris on needle electromyography (EMG) makes the distinction.

A sciatic neuropathy with the involvement of tibial and common peroneal fibres needs to be distinguished from a lumbosacral plexopathy. The superior gluteal nerve, which innervates the gluteus medius (responsible for leg abduction) and tensor fascia lata muscles, arises from the lumbosacral plexus but not the sciatic nerve. Similarly, the inferior gluteal nerve, which innervates the gluteus maximum (responsible for leg extension), arises from the lumbosacral plexus and not the sciatic nerve. The presence of either hip abduction weakness or hip extension weakness in a patient with ankle dorsiflexion and ankle plantar flexion weakness indicates a lumbosacral plexopathy rather than a sciatic neuropathy.[Fig f16-17mjms3106_bc]

## Approaches to Vision Problems

### Approach to Blurry Vision

[Fig f17-17mjms3106_bc]

**Figure 17 f17-17mjms3106_bc:**
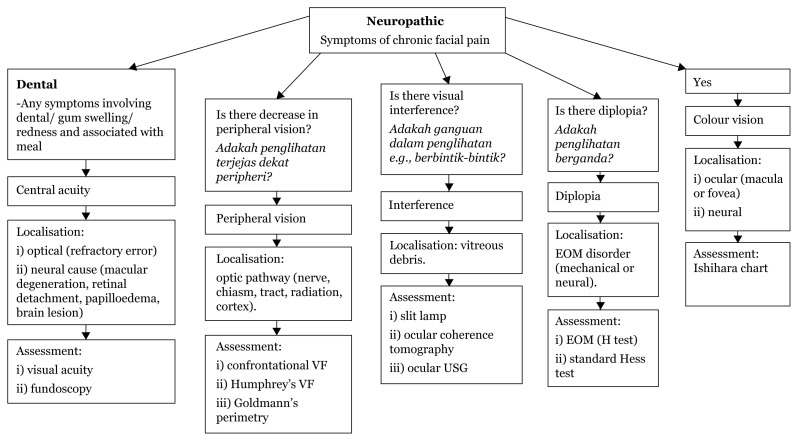
Localisation from visual assessment Note: VF = visual field; USG = ultrasound; EOM = extraocular muscle

### History Taking

First, it is vital to identify a patient’s meaning of blurred vision as follows:

- central acuity: unclear image nearby or far away
- peripheral vision field: decrease in peripheral vision – history of frequent bumping into edge of objects, e.g., closets or scraping the side of a car during parking
- interference with clear image: image is clear but has interference, e.g. floaters or flashes of light
- diplopia: double image – is this horizontal or vertical? Is it corrected by head position adjustment? Is it worse at any gaze?
- colour vision: inability to see colours.

### Blurring of Vision

[Fig f18-17mjms3106_bc]

In clinical examination, it is important to check the eyes and other relevant cranial nerves.

For CN II, it is vital to inspect for any eye proptosis/swelling/haematoma/obvious pulsation. Visual acuity, visual field, pupillary reflexes (relative afferent pupillary defect if present), fundoscopy, and colour vision are required as full CN II examinations.

For a complete eye examination, it is essential to examine CN III, IV, V, and VI as well as check for Horner syndrome. This will aid in localisation.

## Approach to Hearing Impairment

[Fig f19-17mjms3106_bc]

### Examination

#### Subjective

##### Clinical

- Rinne
- Weber
- Schwabach test (comparing patient and examiner hearings)[Table t3-17mjms3106_bc]

##### Pure tone audiometry

- determine type and severity of one’s hearing loss (absolute perceptual threshold of sound, also referred to as peripheral function)

##### Speech audiometry

indicates the ability to understand speech (speech intelligibility and discrimination)

provides direct information on ability to hear and understand of speech

#### Objective

##### Caloric test

- vestibular nuclei superiorly
- horizontal semicircular canals

##### Electronystagmography

- vestibular nuclei sup

##### Acoustic audiometry

- tympanometry

##### Evoked potential

- electrocochleography (ECoG) tests
- vestibular evoked myogenic potentials (VEMPs)
  - vestibular nuclei inferiorly
  - posterior semicircular canals
- auditory brainstem response

## Approaches to Dysphagia

Localisation based on history taking differentiates dysphagia into neurogenic and mechanical types. During further physical examination, based on the types of speech, gag reflex, tongue, and other associated neurological deficits, localisation will produce a few differentials based on location, site, extrapyramidal or even demyelinating disease (Figures 20–22).

### Approach to Dysphagia (History Taking)

**Figure 20:**
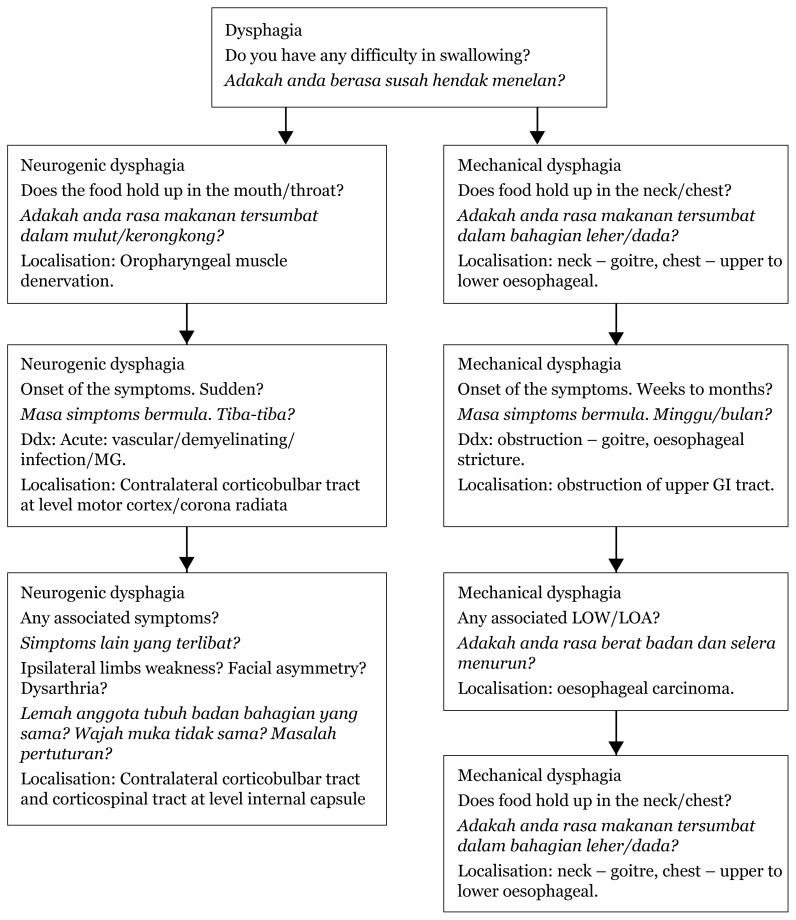
Localisation of neurogenic and mechanical dysphagia Note: MG = Myasthenia gravis; GI = gastrointestinal; LOW = loss of weight; LOA = loss of appetite (LOA)

### Approach to Neurogenic Dysphagia (Physical Examination)

**Figure 21:**
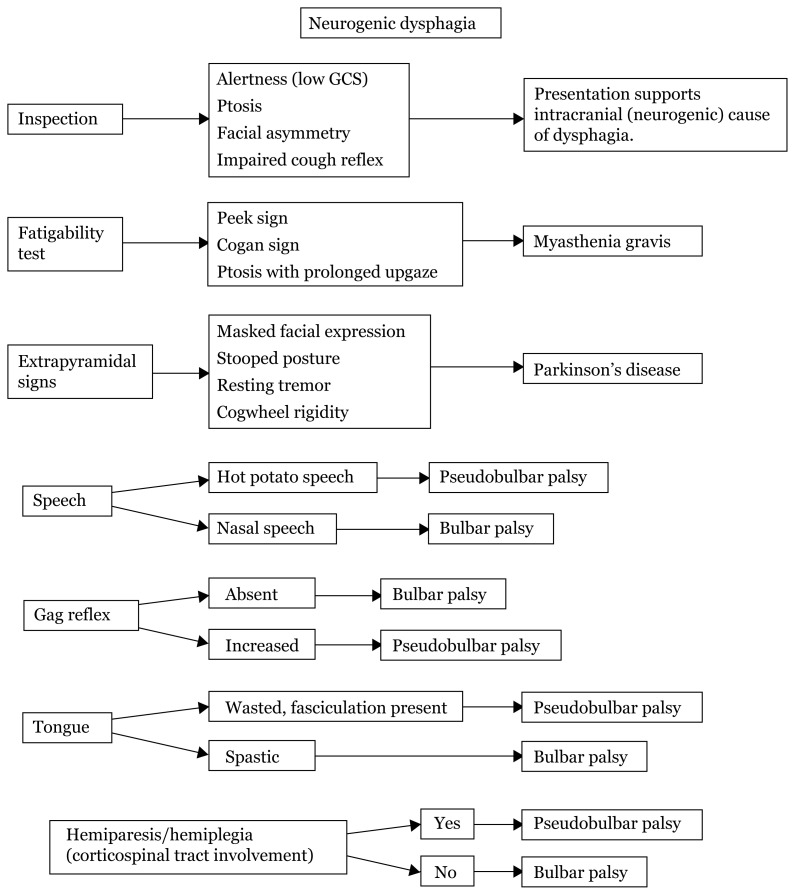
Physical examination of neurogenic dysphagia Note: GSC = Glasgow coma scale

## Approaches to Lower Limbs

When limb weakness is approached in the context of onset of the event, the pattern can be analysed according to the pathology causing the plegia/paresis. In the supratentorial region, areas such as internal capsule, cerebral peduncle (crus cerebri) and pyramid of medulla carry dense corticospinal and corticobulbar tracts. Acute events, such as ischemic infarcts or vascular leaks, in this region lead to devastating hemiplegia contralateral to the lesion due to crossed tracts at the cervical-medullary junction.

Hemiparesis tend to be seen in areas with less dense fibre, such as the centrum semi-ovale, corona radiata, area surrounding the internal capsule (basal ganglia, thalamus and external capsule to the insula region), and lesions, bleeds or infarcts in this area can compress the adjacent internal capsule leading to hemiparesis rather than plegia due to an intact continuity of the tract from the corona radiata.

Acute deficits in weakness mostly depict upper motor neuron features, such as hypertonia, hyper-reflexia and Babinski upgoing. Usually, at the onset, the presentation is mostly reduced tone and areflexia due to sudden loss of control of the particular limb; however, within minutes to hours, the upper motor neuron (UMN) pattern manifests.

Most cranial nerves receive bilateral innervation, which means that only bilateral lesions of the UMN of the cranial nerves will produce deficits. Two exceptions to this are the cranial nerves VII innervating the lower face and XII which receive innervation from contralateral cerebral hemispheres. Identifying deficits of these two cranial nerves helps in determining the level of the UMN lesion.

In addition to weakness, cortical lesions result in other deficits, such as aphasia (if dominant hemisphere is involved), agnosia (loss of ability to perceive a sensation in the presence of a normal sensory pathway), apraxia (inability to perform complex motor tasks) and loss of memory, calculation or spatial orientation. Higher mental functions are preserved in a capsular lesion.[Fig f23-17mjms3106_bc][Fig f24-17mjms3106_bc][Fig f25-17mjms3106_bc][Fig f26-17mjms3106_bc][Fig f27-17mjms3106_bc][Table t4-17mjms3106_bc]

### Approach to Limb Weakness

**Figure 23 f23-17mjms3106_bc:**
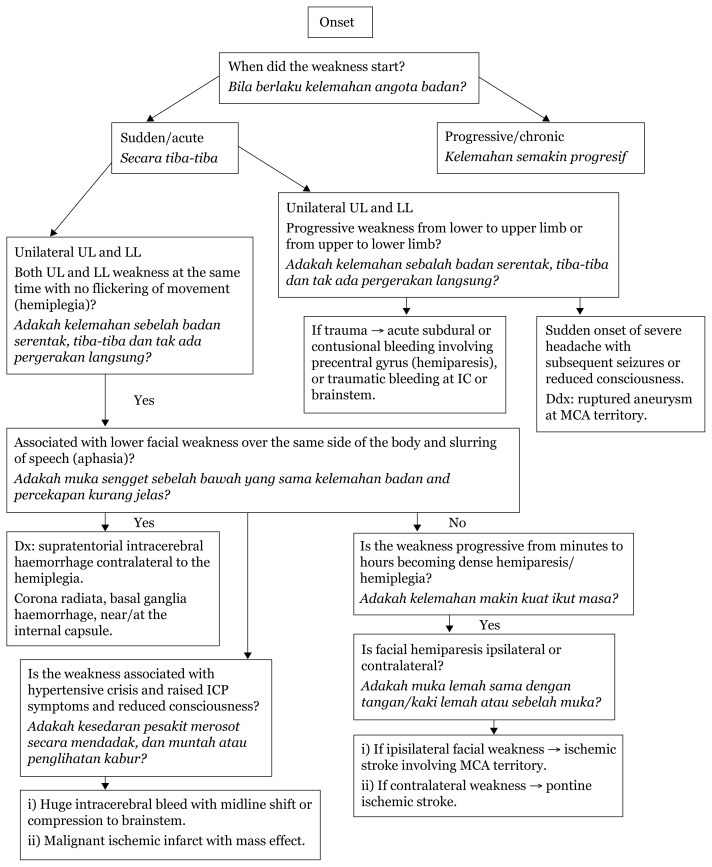
Limb weakness assessment and localisation Note: UL = upper limb; LL = lower limb; IC = internal capsule; ICP = intracranial pressure; MCA = middle cerebral artery

**Figure 24 f24-17mjms3106_bc:**
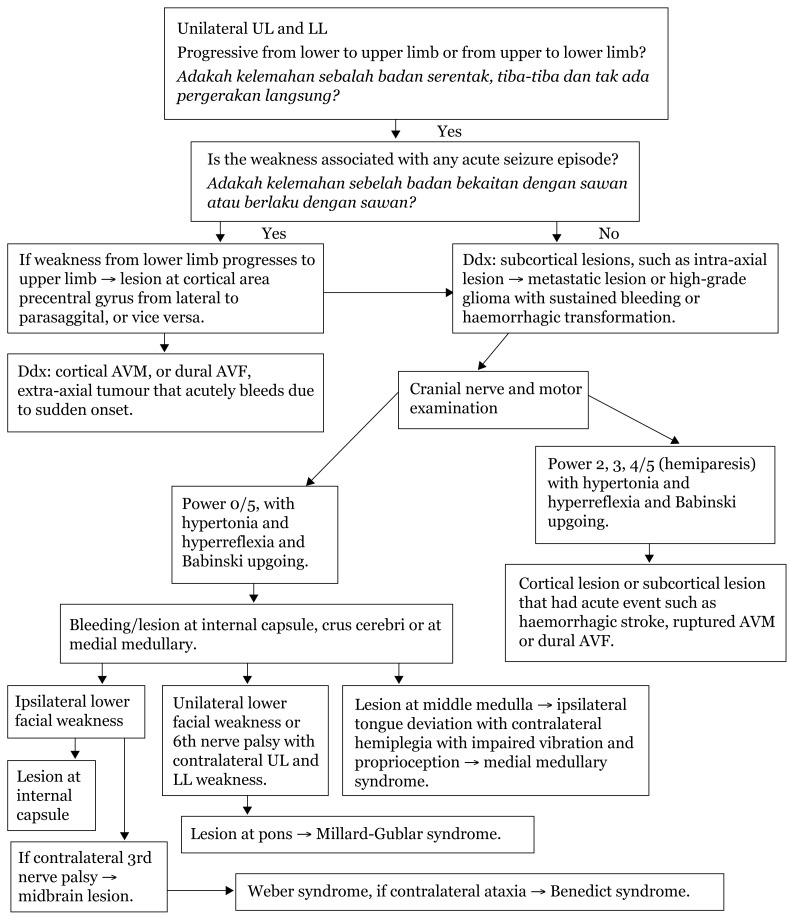
Limb weakness examination and localisation Note: UL = upper limb; LL = lower limb; AVF = arteriovenous fistula; AVM = arteriovenous malformation

**Figure 25 f25-17mjms3106_bc:**
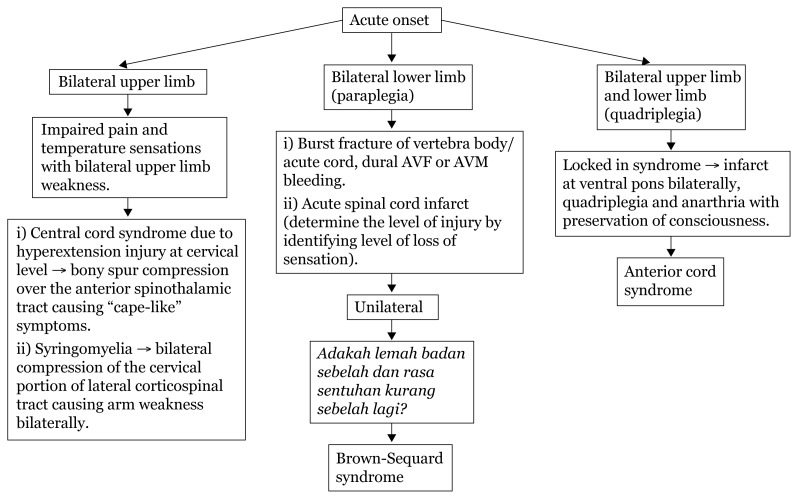
Bilateral limb weakness related to level of pathology Note: AVF = arteriovenous fistula; AVM = arteriovenous malformation

**Figure 26 f26-17mjms3106_bc:**
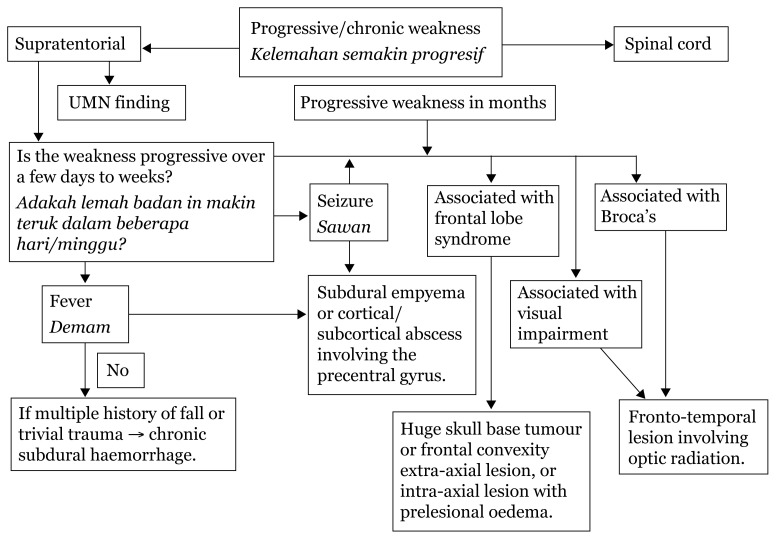
Spinal cord involvement

**Table 5 t4-17mjms3106_bc:** Differentation between extramedullary and intramedullary lesion

Site of Involvement	Outside the cord Extradural or intradural	Within the cord
Symmetry	Asymmetrical	Symmetrical
Spontaneous pain	Radicular, localised distribution, early and important symptom	Funicular, burning type, poorly localised, usually bilateral, often involves large areas of body
Sensory changes	Contralateral loss of pain and temperature ipsilateral loss of proprioception	Dissociative sensory loss (loss of spinothalamic senses and preservation of posterior column senses)
Paraesthesia progression	Ascending	Dscending
LMN involvement	Segmental	Marked and widespread with atrophy and fasciculation
UMN involvement	Prominent	Late, less prominent
Distribution of motor weakness	Cervical lesions cause ipsilateral arm weakness, followed by ipsilateral leg weakness before contralateral side	Cervical lesions can cause unilateral or bilateral upper limb paresis and sparing lower limb in early stages (suspended weakness)
DTR	Increased early	-
Bladder and bowel	Late	Early
Trophic changes	Unusual	Common

Source: De Jong’s Neurological Examination and Brazis Localisation in Clinical Neurology

**Figure 27 f27-17mjms3106_bc:**
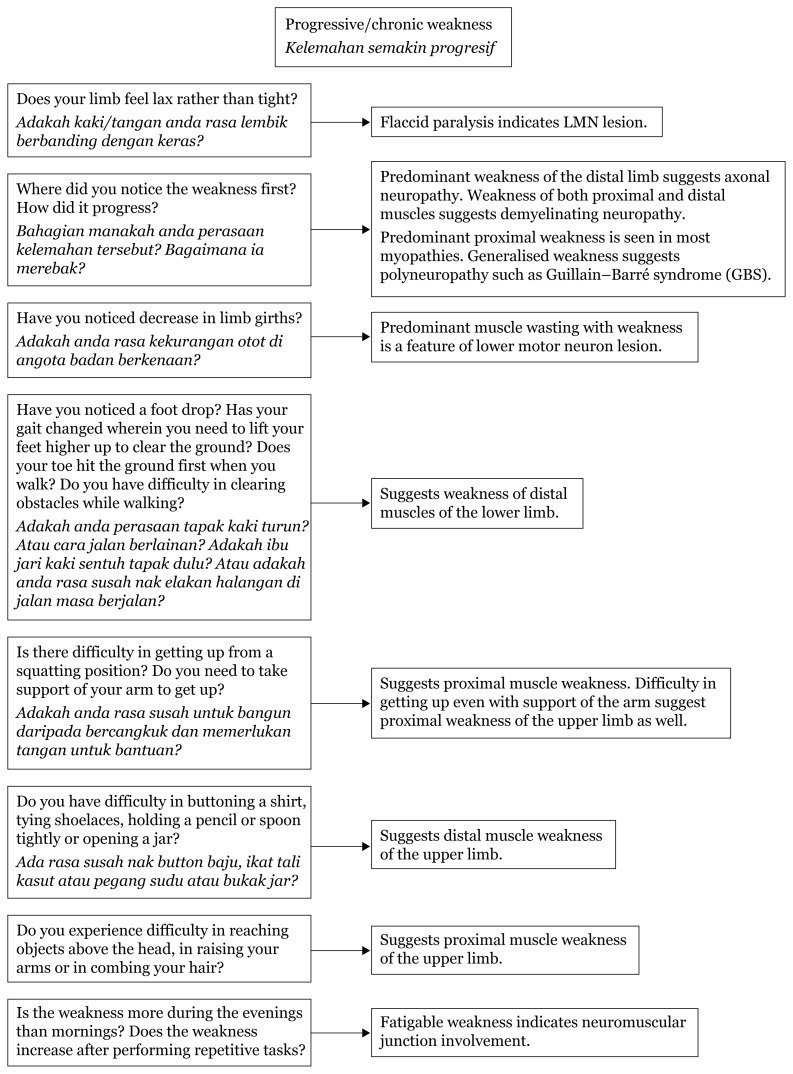
Progressive limbs weakness assessment

## Approaches to Hand Numbness

### Approach to Hand Numbness (Physical Examination)

[Fig f28a-17mjms3106_bc] [Fig f28b-17mjms3106_bc] [Fig f28c-17mjms3106_bc] [Fig f28d-17mjms3106_bc]

**Figure 28a f28a-17mjms3106_bc:**
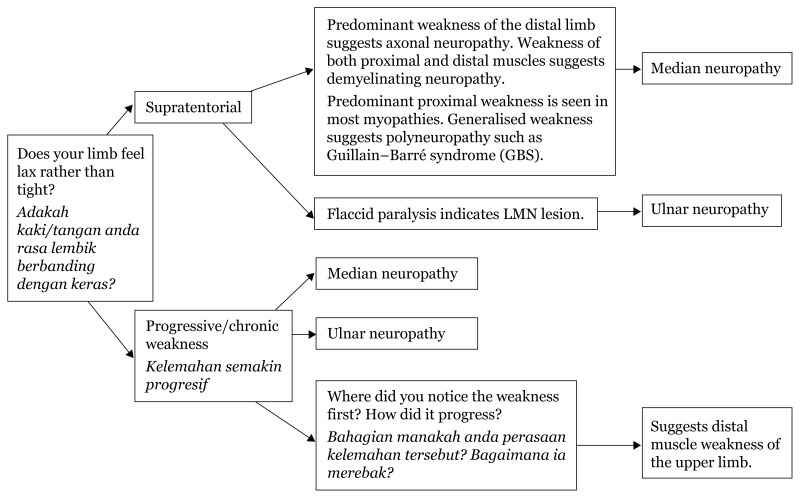
Hand numbness localisation

**Figure 28b f28b-17mjms3106_bc:**
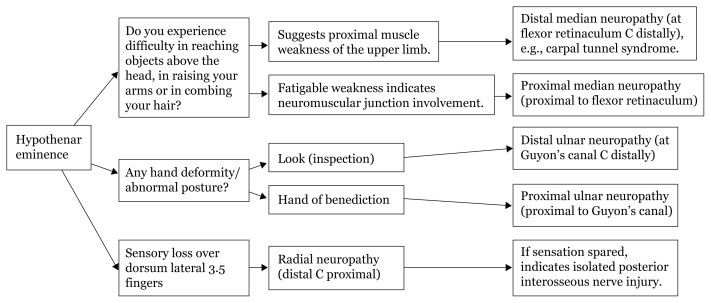
Hand numbness based on level of pathology

**Figure 28c f28c-17mjms3106_bc:**
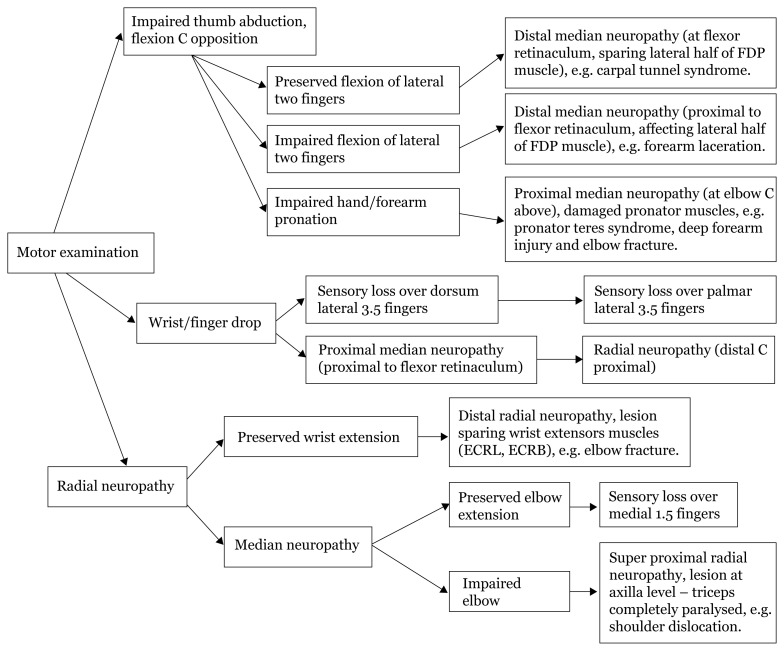
Hand numbness and associated weakness localisation Note: FDP = flexor digitorum profundus; ECRL = extensor carpi radialis longus; ECRB = extensor carpi radialis brevis

**Figure 28d f28d-17mjms3106_bc:**
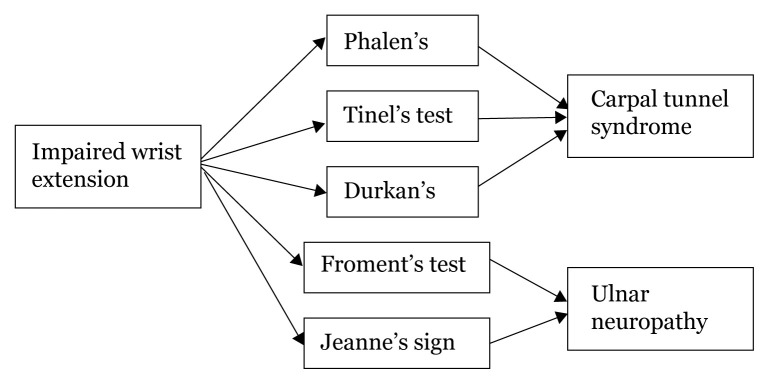
“Special test” for hand examination

### Differential Diagnosis

#### Medial Neuropathy

- Proximal lesion: pronator teres syndrome, supracondylar fracture of humerus.
- Distal lesion: carpal tunnel syndrome, wrist laceration.

#### Ulnar Neuropathy

- Proximal lesion: cubital tunnel syndrome, elbow injuries.
- Distal lesion: wrist laceration.

#### Radial Neuropathy

- Proximal lesion: shoulder dislocation, Saturday night palsy, humeral shaft fracture.
- Distal lesion: elbow injuries.

## Approaches to Urinary Incontinence

[Fig f29-17mjms3106_bc]

**Figure 29 f29-17mjms3106_bc:**
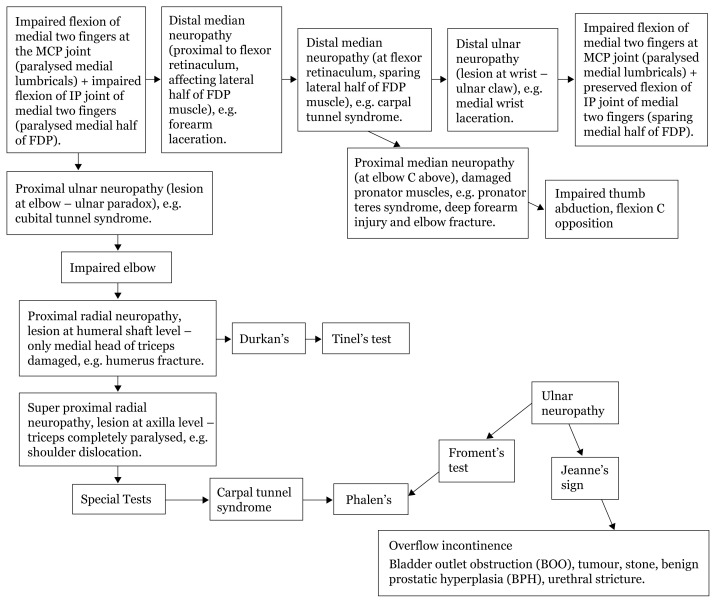
Urinary incontinence localisation

## Bowel Incontinence

[Fig f30-17mjms3106_bc]

Bowel control is achieved by parasympathetic control from the pudendal nerve at levels S2–S4 (16). Lesions causing passive bowel incontinence could stem from the pudendal nerve, or more distally in the spinal cord or at higher centres of the cerebral hemisphere.

Unexplained coexistence of faecal and urinary symptoms in a male should raise suspicion of a spinal lesion particularly in the absence of obstetric trauma and pelvic floor or anal surgery.

**Figure 30 f30-17mjms3106_bc:**
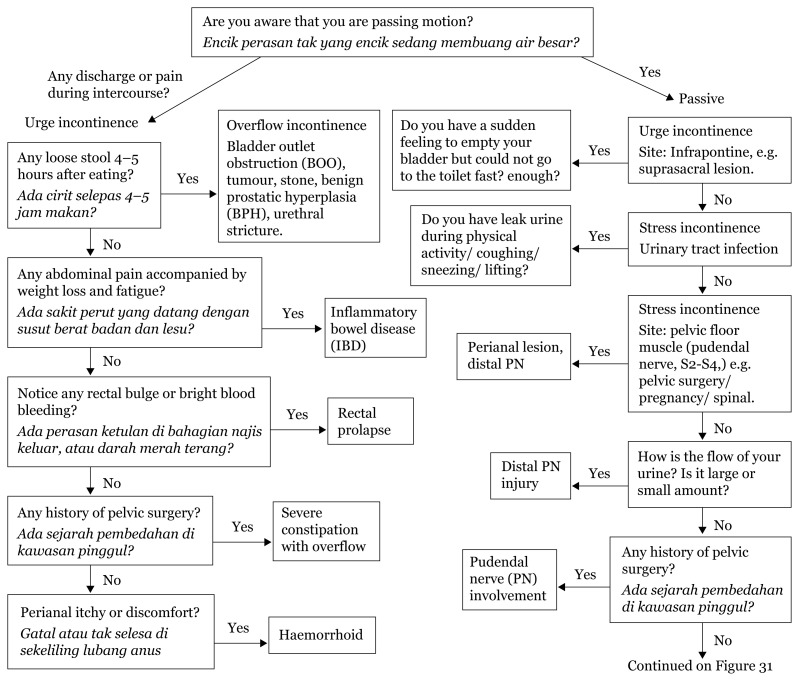
Bowel incontinence localisation

**Figure 31:**
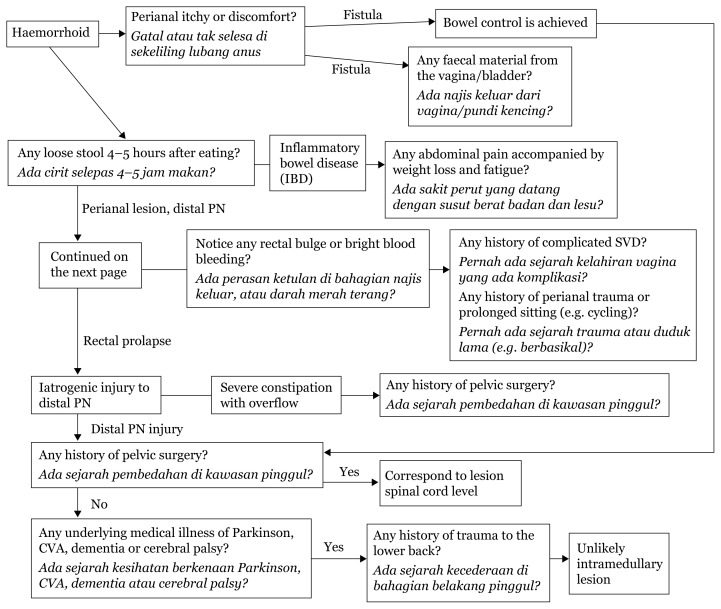
Bowel incontinence localisation (17) Note: SVD = spontaneous vaginal delivery; CVA = cerebrovascular accident

Figures 30 and 31 suggests a comprehensive localisation attempt, from the distal pudendal nerve to the cerebral hemisphere, for possible causes of passive urinary/bowel incontinence. However, physicians should combine all individual patient’s responses to establish a final diagnosis.

## Approaches to Back Lumps

### Spina Bifida Occulta

[Fig f32-17mjms3106_bc]

- Congenital absence of spinous process and variable amounts of lamina.
- No visible exposure of meninges/neural tissues.

### Meningocele

- Congenital defect in vertebral arches with cystic distension of meninges but no abnormality of neural tissue.
- One-third of patients have some neurological deficit.

### Myelomeningocele

- Congenital defect in vertebrae arches with cystic dilatation of meninges and structural or functional abnormality of spinal cord or cauda equina.

### Lipomyelochisis

- Dural spinal dysraphism with lipoma.
- (Intra) dural lipoma.

### Lipomyelomeningocele

- Subcutaneous lipoma that passes through a midline defect in the lumbodorsal fascia, vertebrae arch, dura and merges with an abnormally low tethered cord.
- Fibrolipoma of filum terminale.

## Approaches to Gait Abnormality

### Approach to Gait

Gait is the coordinated movement of the limbs in relation to the body, resulting in the forward movement of the body which is essential for normal walking. This requires the proper functioning of the primary motor and sensory systems, as well as a healthy cerebellum. There are five key functions of walking gait:

- supporting the head, arms and trunk;
- maintaining an upright posture and balance;
- controlling the trajectory of the feet to ensure safe ground clearance and a smooth heel or toe landing;
- generating mechanical energy to sustain or increase forward velocity (concentric muscle actions); and
- absorbing mechanical energy for shock absorption and stability, or to decrease the forward velocity (eccentric muscle actions).[Fig f33-17mjms3106_bc]

### Gait Cycle

#### Phases of the Gait Cycle (New Terms)

Numbers i–v is the stance phase and vi–viii is the swing phase (20):

- initial contact/heel strike;
- loading response/foot flat;
- midstance;
- terminal stance/heel off;
- preswing/toe off;
- initial swing;
- mid swing; and
- late swing/terminal swing.[Fig f34-17mjms3106_bc]

##### Heel Strike (or Initial Contact)

The initial phase of double support, known as the short period, commences as soon as the foot contacts the ground. This phase involves several key movements: a 30° flexion of the hip, full extension in the knee, and a transition of the ankle from dorsiflexion to a neutral (supinated 5°) position before moving into plantar flexion. Following these movements, a 5° knee flexion begins and gradually increases, mirroring the increase in plantar flexion of the heel. The plantar flexion is facilitated by the eccentric contraction of the tibialis anterior, while the extension of the knee is achieved through the contraction of the quadriceps. The flexion is brought about by the contraction of the hamstrings, and the flexion of the hip is a result of the contraction of the rectus femoris.

##### Foot Flat (or Loading Response Phase)

Now, body absorbs the impact of the foot by rolling in pronation. After this, the hip moves slowly into extension, caused by a contraction of the adductor magnus and gluteus maximus muscles. Then, the knee flexes 15° to 20° followed by an increase in ankle plantar flexion of 10°–15°.

##### Midstance

The hip moves from 10° of flexion to extension by contraction of the gluteus medius muscle. This causes the knee to attain maximum flexion and then begins to extend. Afterwards, the ankle becomes supinated and dorsiflexed (5°), which is caused by contraction of the triceps surae muscles. During this phase, the body is supported by one single leg and at this moment the body begins to move from force absorption at impact to force propulsion forward.

##### Heel Off/Terminal Stance

This phase begins when the heel leaves the floor. The bodyweight is divided over the metatarsal heads. Subsequently, about 10°–13° of hip hyperextension occurs, which then goes into flexion. After this, the knee flexes (0°–5°) and the ankle supinates with plantar flexion.

##### Toe Off/Pre-swing

This phase started when hip becomes less extended. The knee is flexed 35°–40° and plantar flexion of the ankle increases up to 20°. Then, the toes leave the ground.

##### Early Swing Phase

This initial swing phase started when hip extends to 10° and then flexes due to contraction of the iliopsoas muscle about 20° with lateral rotation. Afterward, followed by knee flexion to 40°–60° and the ankle goes from 20° of plantar flexion to dorsiflexion, to end in a neutral position.

##### Mid Swing Phase

Hip flexes up to 30° (by contraction of the adductors) and the ankle becomes dorsiflexed due to a contraction of the tibialis anterior muscle. Then, the knee flexes 60° but then extends approximately 30° due to the contraction of the sartorius muscle (caused by the quadriceps muscles).

##### Late Swing Phase/Deceleration

The terminal swing phase begins when hip flexes to 25°–30°. This results in locked extension of the knee and neutral position of the ankle.[Fig f35-17mjms3106_bc]

### Muscle Involvement in Normal Gait

**Figure 35 f35-17mjms3106_bc:**
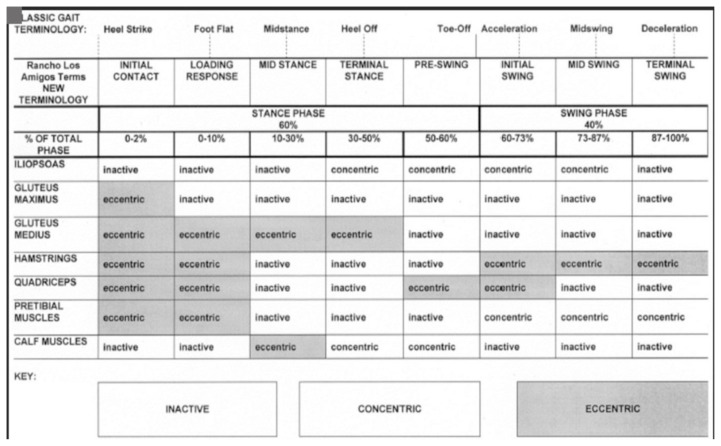
Muscle involvement in normal gait

### Localisation Flow Chart

[Fig f36-17mjms3106_bc] [Fig f37-17mjms3106_bc]

**Figure 36 f36-17mjms3106_bc:**
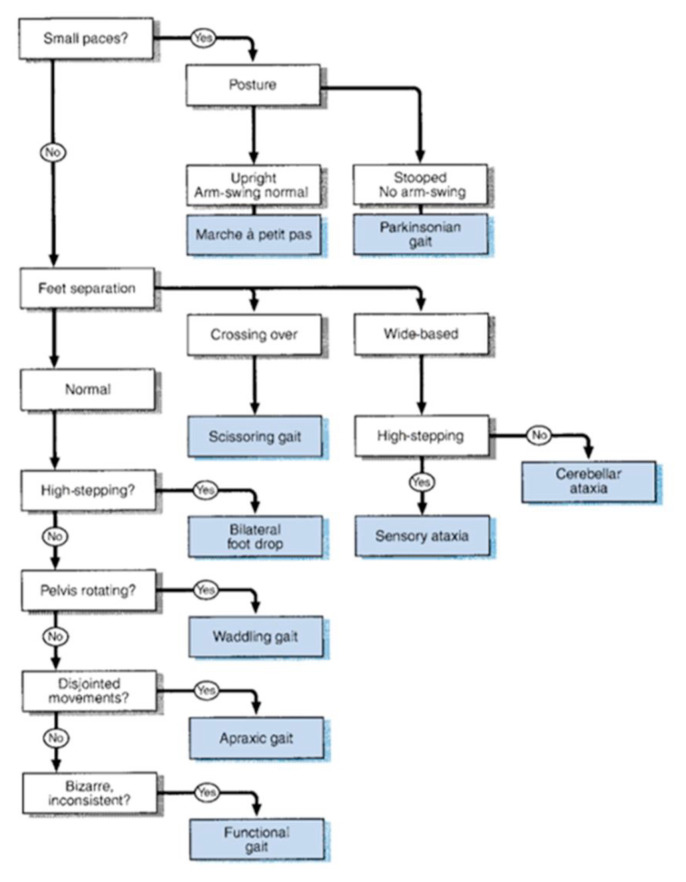
Flow chart of neurological gait examination

**Figure 37 f37-17mjms3106_bc:**
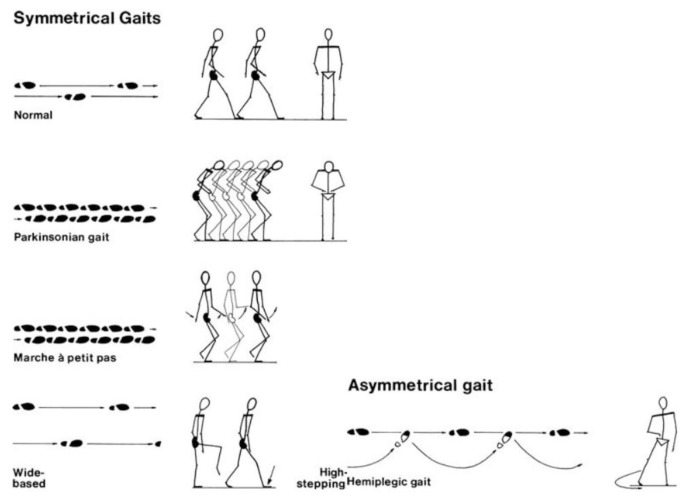
Symmetrical or asymmetrical gait abnormality

## Conclusion

The authors hope that this localisation summary and flow chart for each common neurological signs and symptoms will be helpful in localising and determining the site of lesions in patients with neurological disease.

## Figures and Tables

**Figure 4a f4a-17mjms3106_bc:**
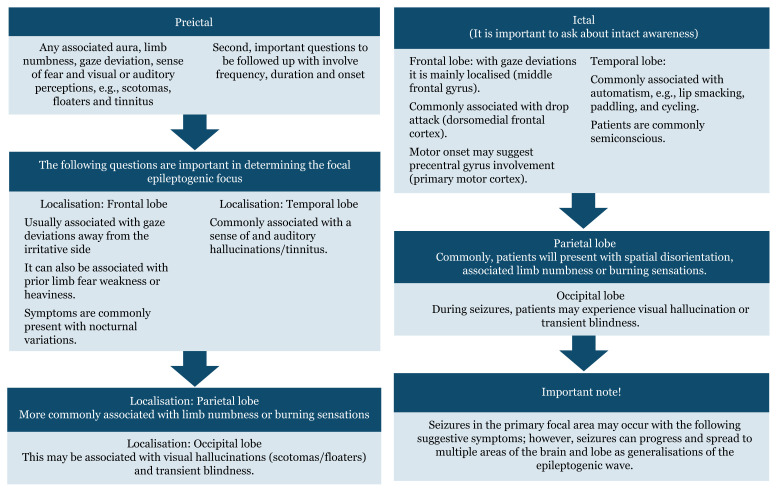
Seizure localisation questions (3–5)

**Figure 4b f4b-17mjms3106_bc:**
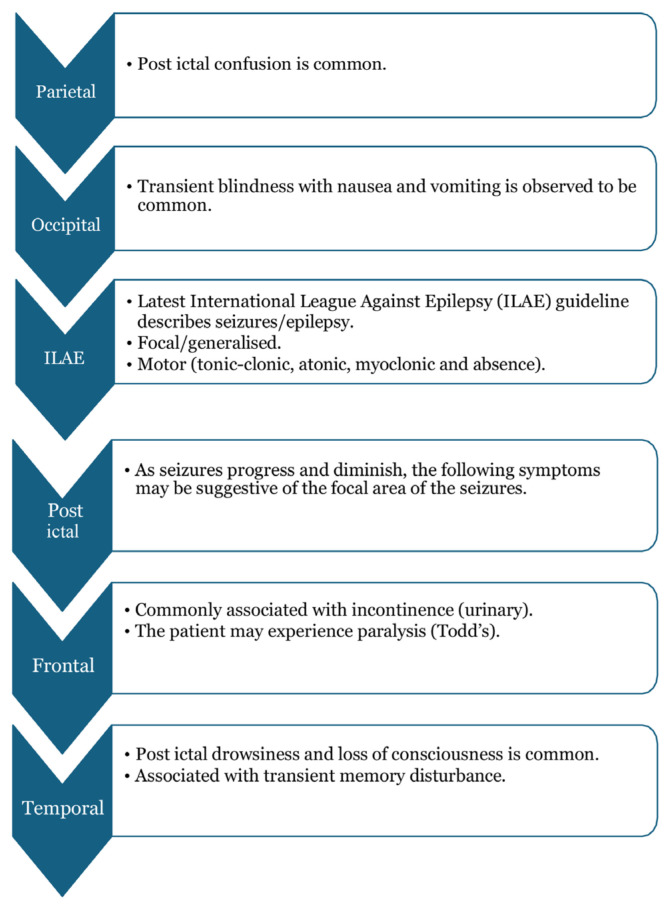
Seizure localisation questions

**Figure 10 f10-17mjms3106_bc:**
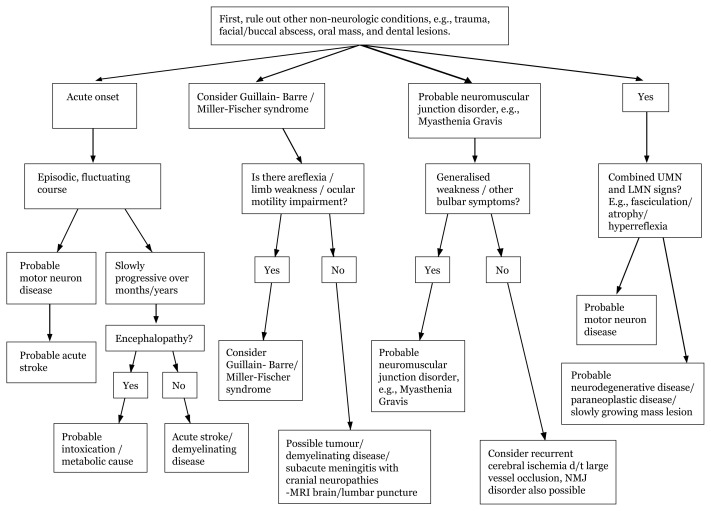
Aetiology dysarthia

**Figure 11 f11-17mjms3106_bc:**
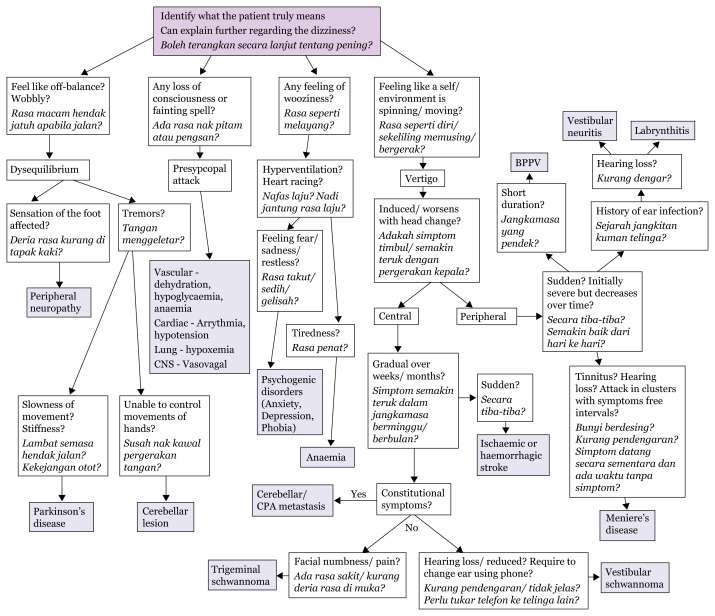
Schematic diagram approach to dizziness

**Figure 15 f15-17mjms3106_bc:**
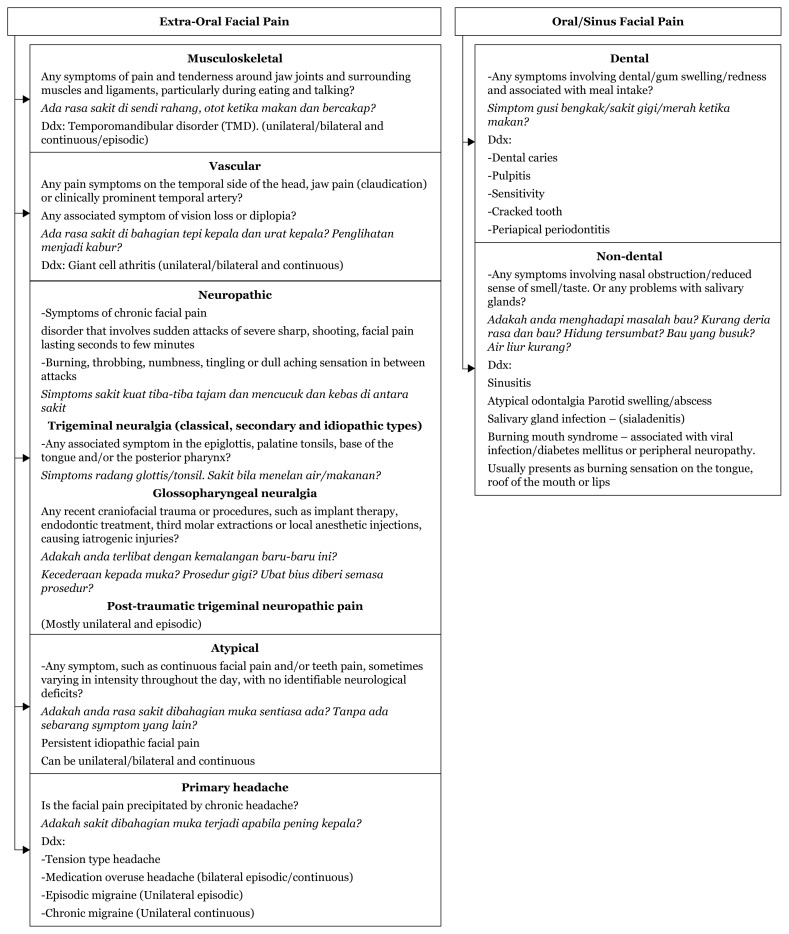
Facial pain localisation (13)

**Figure 16 f16-17mjms3106_bc:**
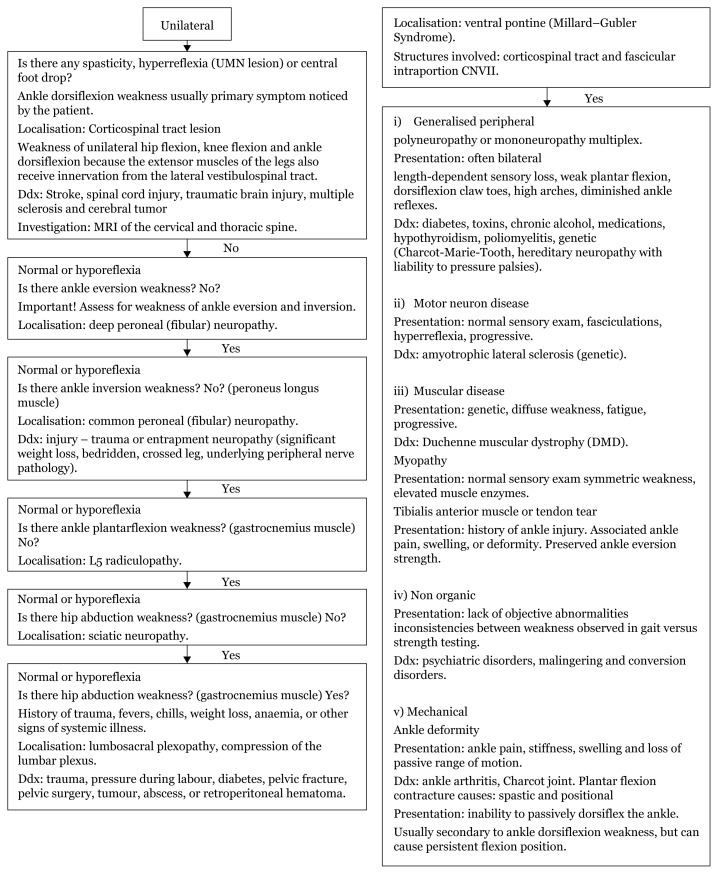
Foot drop localisation

**Figure 18 f18-17mjms3106_bc:**
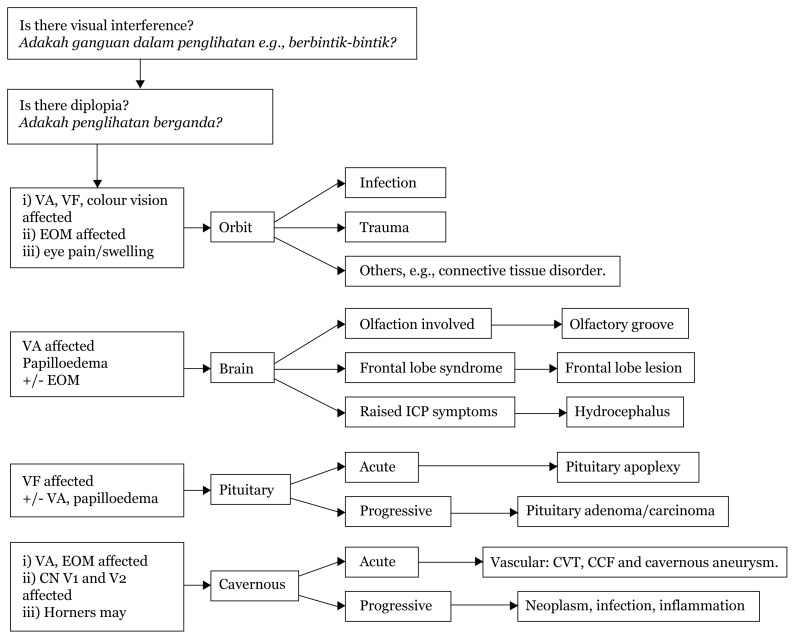
Eye examination and localisation Note: CN = cranial nerve; VA = visual acuity; CVT = cavernous sinus thrombosis; CCF = caroticocavernous fistula

**Figure 19 f19-17mjms3106_bc:**
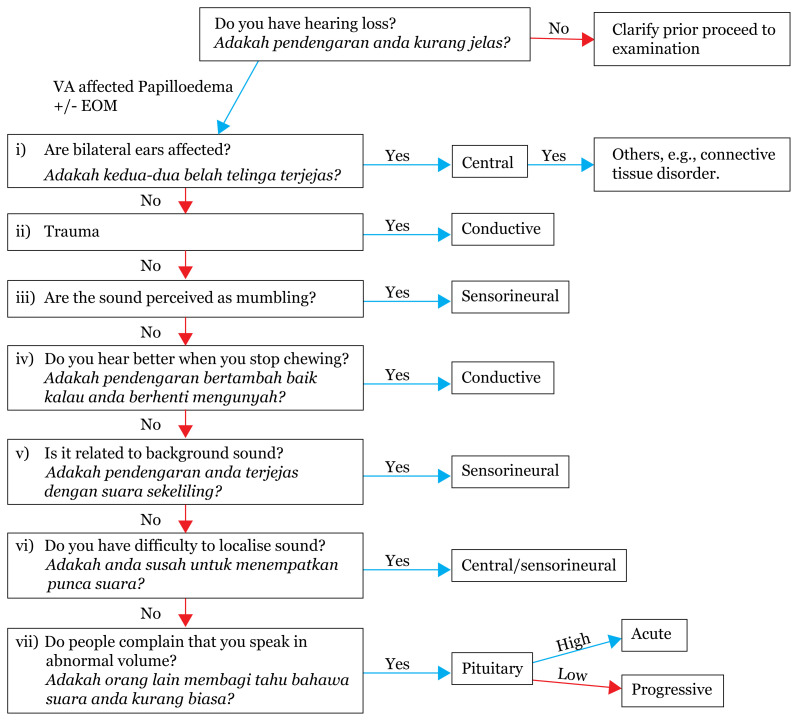
Hearing impairment localisation

**Figure 22:**
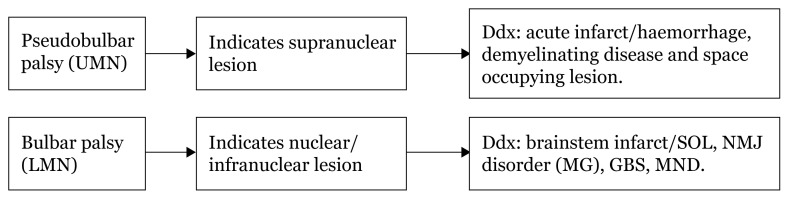
Differential diagnosis of pseudobulbar palsy and bulbar palsy Note: UMN = upper motor neuron; LMN = lower motor neuron; SOL = space occupying lesion; NMJ = neuromuscular junction; MG = myasthenia gravis; GBS = Guillain–Barré syndrome; MND = motor neuron disease

**Figure 32 f32-17mjms3106_bc:**
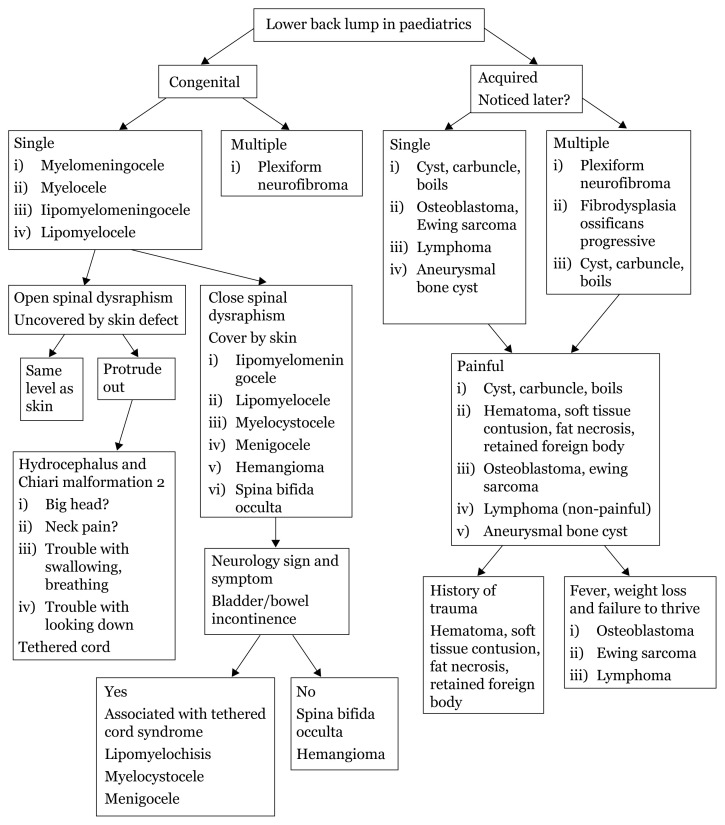
Back lumps history taking (18)

**Figure 33 f33-17mjms3106_bc:**
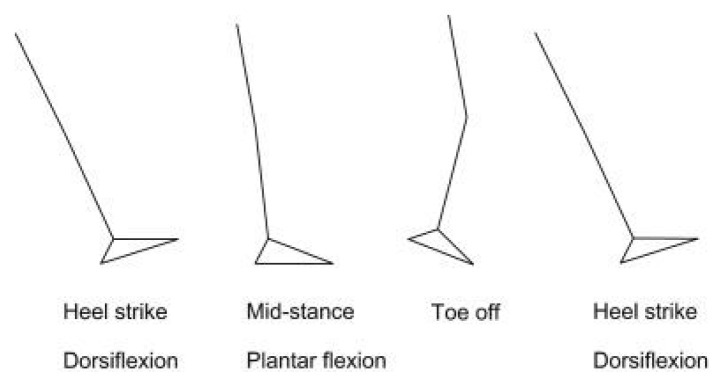
Normal gait step (19)

**Figure 34 f34-17mjms3106_bc:**
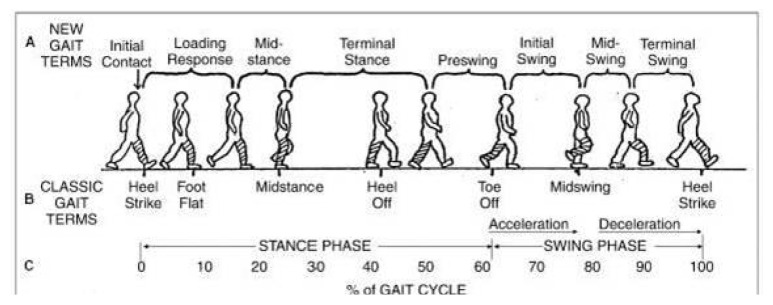
Normal gait cycle and phase (21)

**Table 1:** The optimal cut-off scores for the screening of dementia for M-MMSE-7, M-MMSE-3, M-MMSE-S

	M-MMSE-7	M-MMSE-3	M-MMSE-S
Combined male and female	≤ 21	≤ 18	≤ 17
Male	≤ 23	≤ 22	≤ 19
Female	≤ 19	≤ 18	≤ 18

**Table 2:** Cut-off scores based on education level

Education level (Malaysia)	Cut-off scores
Form 1 or lower	22 or below
Form 2 and above	24 or below
High school graduate (SPM)	25 or below
College or higher (tertiary)	26 or below

**Table 4 t3-17mjms3106_bc:** Summary test

Variable	Auditory acuity	Rinnie test	Weber test
Conductive	Decreased	BC > AC	Lateralises to the abnormal side
Sensorineural	Decreased	AC > BC	Lateralises to the abnormal side

Note: BC = bone conduction; AC = air conduction
